# Web-Based Technology to Improve Disease Knowledge Among Adolescents With Sickle Cell Disease: Pilot Study

**DOI:** 10.2196/15093

**Published:** 2020-01-07

**Authors:** Anjelica C Saulsberry, Jason R Hodges, Audrey Cole, Jerlym S Porter, Jane Hankins

**Affiliations:** 1 Department of Hematology St Jude Children's Research Hospital Memphis, TN United States; 2 Department of Psychology St Jude Children's Research Hospital Memphis, TN United States

**Keywords:** sickle cell anemia, eHealth, transition to adult care

## Abstract

**Background:**

Advancements in treatment have contributed to increased survivorship among children with sickle cell disease (SCD). Increased transition readiness, encompassing disease knowledge and self-management skills before transfer to adult care, is necessary to ensure optimal health outcomes. The Sickle Cell Transition E-Learning Program (STEP) is a public, Web-based, 6-module tool designed to increase transition readiness for youth with SCD.

**Objective:**

The objective of our study was to investigate the participation rate of youth with SCD in STEP and its association with transition readiness.

**Methods:**

This was a single-center, Institution Review Board–approved, retrospective cohort review. A total of 183 youths with SCD, aged between 12 and 15 years, were offered STEP as an adjunct to in-clinic disease education sessions. Participation rate (number of patients who used at least one STEP module divided by those approached) was calculated. The association among the number of STEP modules completed, disease knowledge, and self-management was explored.

**Results:**

Overall, 53 of the 183 approached adolescents completed at least one STEP module, yielding a participation rate in STEP of 29.0%. Of the 53 participants, 37 and 39 adolescents had disease knowledge and self-management confidence rating available, respectively. A positive correlation (*r*=0.47) was found between the number of STEP modules completed and disease knowledge scores (*P*=.003). No association was found between the number of modules completed and self-management confidence ratings. Disease knowledge scores were significantly higher among participants who completed ≥3 STEP modules compared with those who completed <3 STEP modules (*U*=149.00; *P*=.007).

**Conclusions:**

Improvement in disease knowledge in adolescence is critical to ensure the youth’s ability to self-care during the period of transition to adult care. Despite low participation, the cumulative exposure to the STEP program suggested greater promotion of disease knowledge among adolescents with SCD before transfer to adult care.

## Introduction

### Background

Sickle cell disease (SCD) is a genetic disorder characterized by recurrent vaso-occlusive events, chronic pain, and progressive multisystem end-organ damage [1]. In the United States, SCD affects an estimated 100,000 individuals [2,3]. Over the past 5 decades, survivorship into adulthood for US children with SCD has increased to greater than 95%, mostly attributable to newborn screening programs, greater access to care, and use of disease-modifying therapies [4,5]. Increased survivorship to adulthood underscores the adult health care transition period as an important time for individuals with SCD. The transfer period from pediatric to adult care is a high-risk time with an increase in acute health care utilization and acute complications for many chronic diseases [6]. Poor health outcomes during the transfer period for individuals with SCD, including increased acute health care utilization, is complex and may be attributable in part to poor transition readiness [7].

Transition readiness comprises self-management skills and disease knowledge aimed at increasing self-care skills. Individuals with SCD may have deficits in disease knowledge, and both patients and caregivers are interested in interventions to increase disease knowledge and self-management skills [8,9]. In the SCD population, proper transition readiness is correlated with improved transition outcomes and is recognized as an area for intervention to improve health outcomes during the transition period [10,11]. Improved disease knowledge is associated with lower frequency of emergency room visits and higher frequency of outpatient visits among adults with SCD [12].

Interventions that require the physical presence of patients in clinic to deliver the intervention are limited by access to longitudinal care and low adherence to routine clinic visits [13]. The use of electronic devices allows flexibility in utilization beyond the hospital walls, such as their home, school, and other environments. Adolescents with SCD have expressed desire to use mobile health and other Web-based tools as part of their disease management [14]. Over 80% of adolescents and young adults with SCD have access to a computer or mobile phone [15,16]. Among individuals with SCD, Web-based and mobile apps have been shown to increase adherence to medications [16-18]. Taken together, these findings point toward the increasing acceptability of digital interventions for self-management in the SCD population, particularly among adolescents and young adults.

Structured education interventions for patients with SCD before transfer to adult care is limited, especially those focused on transition readiness. A total of 2 studies utilizing electronic platforms among youth with SCD, 1 with mobile technology and 1 with a CD-ROM education game, have demonstrated an increase in disease knowledge [19,20]. Currently, most mobile interventions favor medication adherence, whereas a few apps focus on targeting disease knowledge and self-management skills [21,22]. There is increasing evidence that electronic health (eHealth) interventions improve self-management outcomes for individuals with SCD [14]. The mobile self-management app, iManage, demonstrated feasibility and acceptance among adolescents with SCD [15]. Furthermore, educational handouts were shown to improve preidentified deficits in self-management skills [23].

### Objectives

The Sickle Cell Transition E-Learning Program (STEP) is as a Web-based educational intervention developed to improve SCD knowledge. STEP offers an alternative to the existing disease education interventions as it can be used in any setting and only requires internet access. Thus, our primary objective was to investigate the participation rate in STEP as an intervention to improve transition readiness and to gather preliminary data regarding its correlation with disease knowledge and self-management confidence among adolescents with SCD.

## Methods

### Participant Selection

Individuals with SCD, aged between 12 and 15 years during the first 2 years of STEP implementation, and who were participants of the longitudinal cohort study, Sickle Cell Clinical Research and Intervention Program (SCCRIP) [24], were included. Those who completed 1 or more modules of STEP, in addition to either the disease knowledge or the self-efficacy assessments during pediatric care at St. Jude Children’s Research Hospital were included in the subanalysis of the efficacy of STEP in increasing transition readiness. All participants or their legal guardians (if minors at the time of SCCRIP enrollment) signed the SCCRIP informed consent, which allowed for retrospective data collection for STEP participation.

### Sickle Cell Transition Electronic Learning Program Description

**Figure 1:**
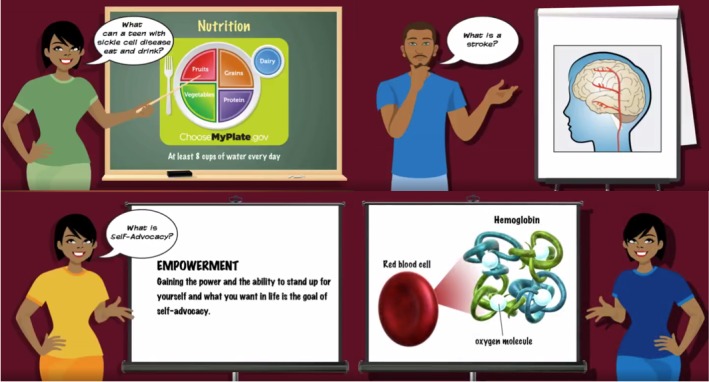
Panel of 4 module videos of the Sickle Cell Transition E-Learning Program (STEP). STEP is a 6-module, Web-based tool designed to increase disease knowledge in patients with sickle cell disease.

The STEP program was developed in 2013 by a nurse from the department of hematology and other SCD program staff as an adjunct to the standard of care in-clinic education sessions offered by the nurse educators. STEP utilizes the electronic learning software Articulate Global Inc, and is an open-access, Web-based tool comprising 6 modules (Figure 1) [25]. It is administered via a tablet device in clinic or at home. Module, video, and quiz contents were developed based on SCD literature and our program’s SCD educational curriculum [21] and includes 6 modules: (1) sickle cell and me; (2) healthy living and SCD; (3) pain, infection, and SCD; (4) other complications of SCD; (5) genes and SCD; and (6) self-advocacy for teens with SCD. Modules 1 and 2 reflect fundamentals of SCD disease knowledge, whereas modules 3 to 6 address advanced topics related to disease complications and necessary transition skills. Each module includes an optional preassessment, educational video of topic, and a scored postassessment (Figure 1). The postassessment scores range between 0 and 100, and STEP participation and score results were recorded after each module completion. Patients with scores of >80 (scale of 1-100) were provided an incentive (eg, small toys and board games). The program did not capture discrete scores within the software, but results were recorded as pass or fail based on scoring 80% or higher.

### Disease Knowledge and Self-Management Assessments

The disease knowledge assessment is delivered at the pediatric clinic visit between the ages of 12 and 15 years and before the participants are transferred to adult care. This knowledge assessment is paper-based and comprises 12 multiple-choice questions serving as a comprehensive review of the adolescent SCD-specific knowledge (Multimedia Appendix 1). The disease knowledge assessment tool was developed by the staff of the Hematology Department at St. Jude Children’s Research Hospital, and it tests knowledge related to the general definition of SCD, complications, and pain (Multimedia Appendix 1) [26]. The scores in the disease knowledge assessment range from 0 to 100, with higher numbers reflecting higher disease knowledge. Self-management confidence was assessed with the Self-Management Skills Checklist (SMSC). The SMSC is a tool modified from the validated Transition Readiness Assessment Questionnaire and assesses perceived disease knowledge and self-management skills (Multimedia Appendix 2) [27,28]. The self-management confidence score is rated on a scale from 1 to 10, such that 1 indicates the lowest and 10 indicates the highest self-confidence levels for self-care.

### Statistical Considerations

The rate of acceptance was calculated by dividing the number of patients who were offered and used at least one STEP module by the total of number of those approached. Disease knowledge scores were calculated as a percentage of correct answers. Owing to the data not being normally distributed, the Spearman rho test was used to examine the association between disease knowledge and self-management scores with the completion of at least one STEP module. The Mann-Whitney test was used to compare the association between disease knowledge scores and the number of STEP modules completed [29]. By design, STEP participation always occurred before the knowledge and self-management assessments. This order was intentional and allowed us to collect preliminary data on the relationship between an early disease-education intervention and later disease knowledge and perceived self-efficacy.

## Results

A total of 183 adolescents between the ages of 12 and 15 years were offered to participate in STEP between 2013 and 2014. Of these, 53 participants (median age 14 years, range 12-15 years; 33/53, 62% male) utilized at least one STEP module, yielding a rate of participation of 29.0%. Most participants had HbSS or HbSβ^0^ thalassemia; all were African American, and the demographic characteristics did not differ among males and females (Table 1). There were no differences in demographics related to race, age, sex, and sickle genotype among the STEP participants (n=53) and nonparticipants (n=130). The median number of STEP modules completed was 3 (range 1-6). All (53/53, 100%), 83% (44/53), 62% (33/53), 43% (23/53), 4% (2/53), and 4% (2/53) of the patients completed 1, 2, 3, 4, 5, and 6 STEP modules, respectively. The time to complete each STEP module was approximately 12 min to 18 min. All modules were completed within 12 months from the first module by those who completed >1 module. All participants scored greater than 80% on the module postassessment test.

**Table 1:** Sickle Cell Transition E-Learning Program participants’ characteristics (N=53).

Characteristics		Statistics
**Sex, n (%)**		
	Male	33 (62)
	Female	20 (38)
**Genotype, n (%)**		
	HbSS/ HbSβ^0^ thalassemia	35 (66)
	HbSC/HbSβ^+^ thalassemia	18 (34)
Race (African American), n (%)		53 (100)
Ethnicity (non-Hispanic), n (%)		53 (100)
**Sickle Cell Transition E-Learning Program** **modules completed**		
	Mean (SD)	2.79 (1.08)
	Median (range)	3.00 (1-6)
**Grouping of modules completed, n (%)**		
	1-2 modules	20 (38)
	3-6 modules	33 (62)

**Figure 2:**
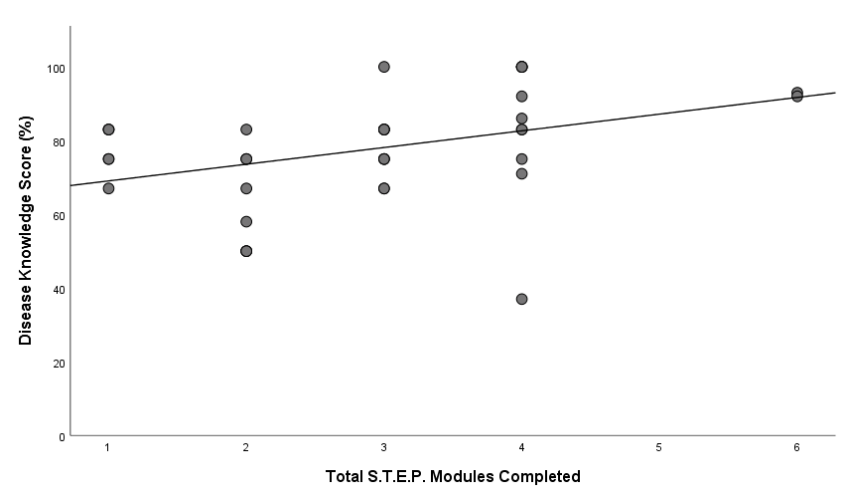
Relationship between the number of Sickle Cell Transition E-Learning Program (STEP) modules completed and the disease knowledge score. A dose-response relationship was found such that greater number of STEP modules completed positively correlated (*r*=0.47; *P*=.003) with disease knowledge scores before transfer to adult care.

Out of the 53 adolescents who completed at least one STEP module, 37 completed the disease knowledge assessment and 39 completed the SMSC before transfer to adult care and comprised the subset for whom preliminary STEP efficacy was tested. Their median age upon completion of the disease knowledge assessment and SMSC was 16 years (range 15-17 years) and 15 years (range 13-17), respectively (Table 2). STEP participation occurred a median of 2.6 and 1.3 years before the disease knowledge assessment and SMSC evaluations, respectively. The median self-management confidence score was 8 (range 5-10) and median knowledge assessment score was 79 (range 37-100). A positive correlation (*r*=0.471) was found between the number of STEP modules completed and the disease knowledge score (*P*=.003; Figure 2). No correlation was found between the number of modules completed and the self-management confidence ratings (*P*=.945). 

The median disease knowledge score was significantly higher among participants who completed ≥3 STEP modules (82.7 [SD 14.68]) compared with those who completed ≤2 modules (69.57 [SD 12.82]; *U*=149.0; *P*=.007). Furthermore, a positive correlation (*r*=0.502) was found between the disease knowledge score and self-management confidence rating (*P*=.005). We repeated the analysis with the 35 participants who had completed modules 1 to 4 only and the results remained the same such that there was a positive relationship between the number of STEP modules and greater disease knowledge (Multimedia Appendix 3). There were no significant differences in STEP scores, knowledge, or SMSC scores by sex. Of the 130 who did not participate in the STEP program, 57 completed the disease knowledge assessment before they transitioned to adult care. When the 57 youths who did not participate in STEP were compared with the 37 who did, there were no statistical differences with regard to demographics (sex, sickle cell genotype, or disease knowledge score).

**Table 2:** Transition readiness scores of Sickle Cell Transition E-Learning Program participants (N=53).

Score characteristics		Values
**Age at first STEP^a^ module (years)**		
	Mean (SD)	14.07 (1.27)
	Median (range)	14.19 (12-15)
**Disease knowledge score**		
	STEP participants with score, n	37
	Mean (SD)	77.53 (14.69)
	Median (range)	79 (37-100)
**Age at disease knowledge score (years)**		
	Mean (SD)	16.0 (0.263)
	Median (range)	16 (15-17)
**Self-management confidence score**		
	STEP participants with score, n	39
	Mean (SD)	8.05 (1.58)
	Median (range)	8 (5-10)
**Age at self-management confidence score (years)**		
	Mean (SD)	15.18 (0.756)
	Median (range)	15 (13-17)
**Time between administration of disease knowledge and self-management assessments (months)**		
	Mean (SD)	11.5 (7.34)
	Median (range)	12 (5-38)

^a^STEP: Sickle Cell Transition E-Learning Program.

## Discussion

### Principal Findings

Disease knowledge is an important component of transition readiness and low knowledge may serve as a barrier to adequate transition outcomes. Our findings suggest that Web-based technology may improve disease knowledge among adolescents with SCD. Greater transition readiness may mitigate the deterioration of health outcomes for patients with SCD during the transition period; however, there is a paucity of studies investigating the role of structured education interventions to improve transition readiness. Few reported structured education programs for adolescents with SCD have utilized eHealth technologies, although a paper-based format to deliver disease education demonstrated feasibility [23]. In addition, new apps in development offer disease education and self-management strategies but have not yet been formally tested [15]. STEP offers an alternative to existing education interventions as it encompasses basic and advanced SCD knowledge and self-management skills necessary for transition to adult care and can be used on any device with internet connection and without the need for an app.

STEP is a readily accessible free resource that can be used by hematology clinic staff as a primary or adjunct intervention to educate youth in preparation for transition to adult care. The easy accessibility of STEP allows for its use in mobile devices or desktops, facilitating its adoption outside the clinic. Furthermore, assessment for each module allows clinical staff to monitor individual progress and identify potential gaps in knowledge. Although we were able to demonstrate a relationship between STEP participation and disease knowledge retention, we did not find a relationship between participation in the program and higher self-management confidence ratings. However, we did find a relationship between disease knowledge and self-management confidence scores, suggesting that increased disease knowledge may be correlated with greater self-management skills. Furthermore, we only included 1 aspect of the SMSC in our analysis, the confidence rating, which may have reduced our sensitivity to detect any association between STEP participation and perceived self-management. It is possible that there are other aspects of self-management that may have been associated with STEP exposure; however, they were not measured in our study.

### Limitations

There were several limitations. The study was a small single-center retrospective cohort study; thus, our findings may not be representative of other adolescent SCD populations. In addition, STEP is embedded within our comprehensive transition program (Saulsberry et al, forthcoming) and the impact of education delivered throughout other aspects of the transition program between time of STEP participation and time of transition readiness assessments is unknown. STEP preassessment and postassessment score documentation was limited as scores were recorded as pass or fail and could not be correlated with comprehensive disease knowledge assessment. Although the rate of participation was low, our sample size was small and limited by completion of both the disease education assessment and the SMSC. This limitation could be because of poor compliance with routine care visits, which is high for many adolescents with chronic diseases [13], and low completion of both the knowledge and the SMSC assessments. It is unclear as to why we did not observe a difference in disease knowledge between the groups participating in STEP and those not participating in STEP, but small sample size in the STEP participant group, a nonrandomized design, and possible differential exposure to other education methods are all plausible reasons. Nonparticipation in the program was not formally measured; therefore, we are unable to provide qualitative reasons for nonparticipation. However, lack of time to complete the STEP modules or lack of interest are possible explanations.

### Future Work

We plan to qualitatively assess engagement in STEP as a future phase in our research. In addition, we plan to formally investigate satisfaction (patient and medical provider) and perceived benefit of STEP and the relationship among the use of STEP, educational outcomes (eg, achievement and attainment), and clinical outcomes during adult care (eg, medication adherence or compliance with medical visits as adults). As STEP can be used outside the clinic, future work will also capture how frequently it is being accessed outside the clinic environment. Finally, future work needs to be done to rule out selection bias as we cannot be certain that those who chose not to participate in the STEP intervention have different educational needs or motivation levels than those who chose to participate.

### Conclusions

In summary, STEP, a Web-based SCD educational tool, had a suboptimal engagement rate; however, preliminary findings suggested that greater STEP use promoted greater disease knowledge among adolescents with SCD. STEP can potentially be used as an intervention to provide SCD education among adolescents with SCD, with the goal of improving their transition readiness. Future work includes investigating the relationship of STEP with other clinical, educational, and behavioral outcomes and strategies to improve participation.

## Appendix

Multimedia Appendix 1 The disease knowledge assessment is paper-based and comprises 12 multiple-choice questions serving as a comprehensive review of adolescent sickle cell disease knowledge.

Multimedia Appendix 2 The Self-Management Skills Checklist for teens is a tool modified from the validated Transition Readiness Assessment Questionnaire and assesses perceived disease knowledge and self-management skills.

Multimedia Appendix 3 Relationship between the number of Sickle Cell Transition E-Learning Program modules completed and disease knowledge score among participants that completed 1-4 modules.

## Abbreviations

eHealth: electronic health
SCCRIP: Sickle Cell Clinical Research and Intervention Program
SCD: sickle cell disease
SMSC: Self-Management Skills Checklist
STEP: Sickle Cell Transition E-Learning Program
