# Supplementary material for: Web-Based Technology to Improve Disease Knowledge Among Adolescents With Sickle Cell Disease: Pilot Study
Source: JMIR Pediatr Parent. 2020 Jan 7;3(1):e15093. doi: 10.2196/15093 (PMC6996770; doi:10.2196/15093)
Supplement: Multimedia Appendix 1 [file pediatrics_v3i1e15093_app1.pdf]

Phase V1II Teaching
Comprehensive Review

1. The hemoglobin is measured in a lab test called _______ _______ _______

    Complete blood count
    Sickle cell test
    White blood test

2. Sickle cell disease is a blood disorder that affects the __________ blood cells.
    Red
    White
    Nerve

3. ____________ is pneumonia along with one or more of the following symptoms: fever; chest pain; coughing; wheezing; or shortness of breath.
    Acute Chest Syndrome
    Stroke
    Osteomyelitis

4. Pain has many different causes; but the most common cause of sickle cell pain is due to
    Sickling of the red blood cells
    Drinking plenty of water
    Exercising

5. __________ is damage to the brain due to lack of oxygen supply. Symptoms can include paralysis in part of the body; changes in visions; seizures; and/ or changes in speech
    Stroke
    Avascular necrosis (AVN)
    Osteomyelitis

6. Red blood cells contain _____________.

    Hemoglobin
    Water
    Nutrients

7. ________ is damage to the eye caused by sickle cells blocking the eye vessels. This requires an eye exam every year for patients with Hemoglobin SC disease and every 2 years for all others sickle cell patients.
Retinopathy
Gallstones
Avascular necrosis

8. Name the most common sign of an infection.
Fever
Stomach pain
Nausea
Runny nose

9. Hemoglobin carries __________ throughout the body. Answer
Oxygen
Food
Water

10. __________ is an infection in the bone and symptoms include fever; swelling; heat; and/or soreness to the area.
Osteomyelitis
Gallstones
Acute Chest Syndrome

11. ___________ is caused by loss of blood (oxygen) supply to the bone which can result in painful bone to bone contact
Avascular necrosis (AVN)
Stroke
Retinopathy

12. Management for sickle cell pain at home includes which of the following
Drinking water
Applying heat to the area
Getting rest
Taking an anti inflammatory such as ibuprofen or naproxen
All of the above
