# Supplementary material for: Web-Based Technology to Improve Disease Knowledge Among Adolescents With Sickle Cell Disease: Pilot Study
Source: JMIR Pediatr Parent. 2020 Jan 7;3(1):e15093. doi: 10.2196/15093 (PMC6996770; doi:10.2196/15093)
Supplement: Multimedia Appendix 2 [file pediatrics_v3i1e15093_app2.pdf]

Name:______________________________ MRN: ____________ Date: ______________

Self-Management Skills Checklist for Teens

Directions: **Below is a list of skills that teens use to manage their sickle cell disease. Please circle the number that best describes your child for each question.**

①	②	③	④	⑤
**No, I do not do this.**	**No, but I am learning how to do this.**	**Yes, I have started doing this when I am reminded.**	**Yes, I always do this when I am reminded.**	**Yes, I always do this when I need to.**

**1.** Can you name all of your medicines?	1	2	3	4	5
**2.** Can you find information about sickle cell disease if you need it (like at the doctor's office, on the internet, or in the community)?	1	2	3	4	5
**3.** Can you find information about your educational options if you need it?	1	2	3	4	5
**4.** Do you ask your doctor or medical team questions during clinic appointments?	1	2	3	4	5
**5.** Do you use strategies other than pain medicines to manage your pain (like deep breathing, distraction, imagery)?	1	2	3	4	5
**6.** Do you take your medicines when you are supposed to?	1	2	3	4	5
**7.** Do you refill your pain medicines on your own?	1	2	3	4	5
**8.** Do you complete a medical history form on your own?	1	2	3	4	5
**9.** Do you take part in activities to stay healthy like exercising and eating healthy foods?	1	2	3	4	5
**10.** Do you find someone to talk to if you feel sad, down, anxious, or angry?	1	2	3	4	5
**11.** Do you talk to your school or medical team about your education and the future?	1	2	3	4	5

Name:_______________________ MRN: _______________ Date: _______________

	① No, I don't know.	② No, but I am learning.	③ Yes, I know.
**1.** Do you know the type of sickle cell disease you have?	1	2	3
**2.** Do you know about complications related to sickle cell disease (like stroke, retinopathy, and bone infection)?	1	2	3
**3.** Do you know how to manage mild to moderate pain episodes at home?	1	2	3
**4.** Do you know how to prevent a pain crisis?	1	2	3
**5.** Do you know when to go to the hospital if you can't manage your symptoms at home?	1	2	3
**6.** Do you know how often you need to come to your clinic appointments?	1	2	3
**7.** Do you know which insurance plan you have?	1	2	3
**8.** Do you know how to find a doctor (not at St. Jude) who is covered by your insurance plan?	1	2	3
**9.** Do you know how to protect yourself from unplanned pregnancy and sexually transmitted diseases (STDs)?	1	2	3
**10.** Do you know the effects of tobacco, street drugs, and alcohol on your body?	1	2	3

On a scale from 0 to 10, please circle one number that best describes how confident you feel about managing your illness on your own.

0 1 2 3 4 5 6 7 8 9 10

Not confident — Very confident

Biomedical Communications 9/14 (AK7600)
