# Supplementary figures and images for: Web-Based Technology to Improve Disease Knowledge Among Adolescents With Sickle Cell Disease: Pilot Study
Source: JMIR Pediatr Parent. 2020 Jan 7;3(1):e15093. doi: 10.2196/15093 (PMC6996770; doi:10.2196/15093)

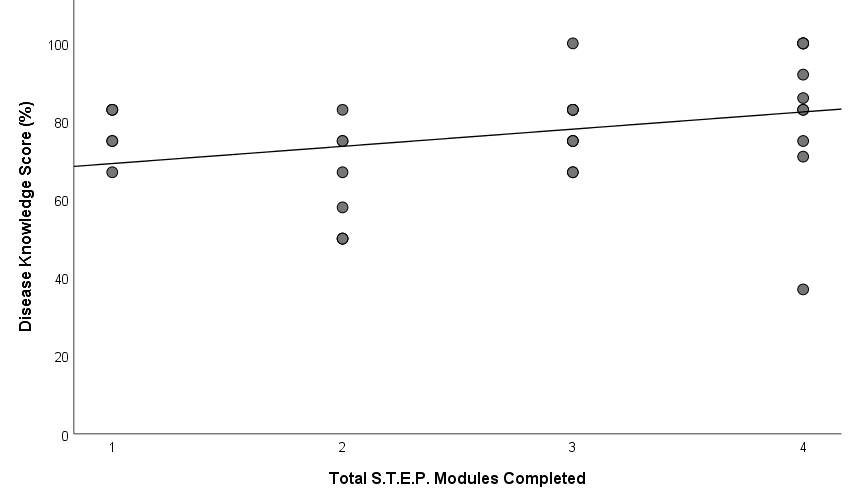

Supplement: Multimedia Appendix 3 [file pediatrics_v3i1e15093_app3.png]
